# Supplementary material for: Phenotypic and Genetic Heterogeneity of Adult Patients with Hereditary Spastic Paraplegia from Serbia
Source: Cells. 2022 Sep 8;11(18):2804. doi: 10.3390/cells11182804 (PMC9497238; doi:10.3390/cells11182804)
Supplement: Supplementary file 1 [file cells-11-02804-s001.zip › cells-1878170-supplementary.pdf]

Supplemental Table S1. Detailed clinical findings of Serbian patients with genetically confirmed SPG4.

Patient	Family	Gender	Age at onset	Age	Mobility	LL	UL	Sphincter dysfunction	Sensibility impairment	Additional features	NCS	SSEP	Brain MRI	Spine MRI
**HSP4**	**1**	male	21-40	44	abnormal, but no aids	hyperreflexia, weakness	normal	bowel and bladder	vibration in LL	none	normal	normal	normal	normal
**HSP9**	**2**	male	6-10	39	abnormal, but no aids	hyperreflexia, weakness	hyperreflexia	normal	normal	foot deformities, dysarthria	normal	normal	normal	normal
**HSP10**	**2**	male	6-10	37	abnormal, but no aids	hyperreflexia, weakness	hyperreflexia	normal	normal	dysarthria	normal	abnormal from LL and UL	single WMHL	normal
**HSP11**	**3**	female	21-40	60	abnormal, but no aids	hyperreflexia, weakness, distal muscle atrophy	hyperreflexia	bladder	vibration and touch in LL	none	normal	normal	normal	normal
**HSP12**	**3**	male	11-20	38	abnormal, but no aids	hyperreflexia	normal	normal	normal	epilepsy	not done	normal	hippocam pal sclerosis	not done
**HSP24**	**4**	female	21-40	40	abnormal, but no aids	hyperreflexia	hyperreflexia	norrmal	normal	none	not done	normal	normal	normal
**HSP32**	**5**	female	21-40	34	abnormal, but no aids	hyperreflexia, weakness	hyperreflexia	normal	vibration in LL	none	normal	not done	pineal gland cyst	normal
**HSP33**	**6**	female	41-60	48	abnormal, but no aids	hyperreflexia, weakness	hyperreflexia	bladder	vibration in LL	none	not done	not done	not done	normal
**HSP42**	**7**	female	41-60	47	abnormal, but no aids	hyperreflexia, weakness	hyperreflexia	bowel and bladder	vibration in LL	none	normal	not done	small WMHLs	normal
**HSP52**	**8**	male	6-10	42	abnormal, but no aids	hyperreflexia, weakness, distal muscle atrophy	hyperreflexia	normal	normal	none	normal	normal	normal	normal
**HSP68**	**9**	male	21-40	42	abnormal, but no aids	normal	hyperreflexia	normal	normal	none	not done	not done	smal WMHLs	normal

**HSP80**	**10**	female	21-40	43	abnormal, but no aids	hyperreflexia, weakness	hyperreflexia, mild distal weakness	bladder and bowel	vibration in LL	mild cerebellar ataxia	normal	not done	small WMHLs	normal
**HSP81**	**10**	male	<1	33	abnormal, but no aids	hyperreflexia, weakness	hyperreflexia	bladder and bowel	vibration in LL	deafness, foot deformities	normal	not done	retrocerebellar cyst	normal
**HSP94**	**4**	male	21-40	24	normal	hyperreflexia	hyperreflexia	normal	normal	none	not done	not done	normal	normal
**HSP112**	**11**	female	41-60	44	abnormal, but no aids	asymmetric hyperreflexia, weakness	normal	normal	normal	mild dysarthria, mild cerebellar signs	normal	not done	WMHLs in cerebellum, mild cerebellar atrophy	normal
**HSP114**	**12**	female	>60	72	abnormal, but no aids	hyperreflexia	hyperreflexia	bladder	normal	foot deformities, mild cerebellar ataxia	normal	not done	normal	normal

LL – lower limbs, UL – upper limbs, NCS – nerve conduction studies, SSEP – somatosensory evoked potentials, MRI – muscle resonance imaging, WMHL – white matter hyperintensity lesions
